# Pharmacological Inhibition of Fatty Acid-Binding Protein 4 (FABP4) Protects Against Rhabdomyolysis-Induced Acute Kidney Injury

**DOI:** 10.3389/fphar.2018.00917

**Published:** 2018-08-08

**Authors:** Rongshuang Huang, Min Shi, Fan Guo, Yuying Feng, Yanhuan Feng, Jing Liu, Lingzhi Li, Yan Liang, Jin Xiang, Song Lei, Liang Ma, Ping Fu

**Affiliations:** ^1^Kidney Research Laboratory, Division of Nephrology, West China Hospital of Sichuan University, Chengdu, China; ^2^Core Facility of West China Hospital, Chengdu, China; ^3^Laboratory of Clinical Pharmacology, West China Hospital of Sichuan University, Chengdu, China; ^4^Department of Pathology, West China Hospital of Sichuan University, Chengdu, China

**Keywords:** acute kidney injury, rhabdomyolysis, fatty acid-binding protein 4, endoplasmic reticulum stress, inflammation

## Abstract

Acute kidney injury (AKI) is a common and potentially life-threatening complication. Studies confirmed that circulating FABP4 depended on renal function of AKI patients. In our previous study, FABP4 was involved in the pathogenesis of I/R-induced AKI. However, the function of FABP4 in rhabdomyolysis-induced AKI remained poorly understood. In the study, we further investigated the effect of FABP4 in a murine model of glycerol injection-induced rhabdomyolysis. Following glycerol injection, the mice developed severe AKI as indicated by acute renal dysfunction and histologic changes, companied by the increased FABP4 expression in the cytoplasm of tubular epithelial cells. Pharmacological inhibition of FABP4 by a highly selective inhibitor BMS309403 significantly reduced serum creatinine level, proinflammatory cytokine mRNA expression of tumor necrosis factor-α, interleukin-6, and monocyte chemoattractant protein 1 as well as attenuated renal tubular damage in glycerol-injured kidneys. Oral administration of FABP4 inhibitor also resulted in a significant attenuation of ER stress indicated by transmission electron microscope analysis and its maker proteins expression of GRP78, CHOP, p-perk, and ATF4 in kidneys of AKI. Furthermore, BMS309403 could attenuate myoglobin-induced ER stress and inflammation in renal proximal tubular epithelial cell line (HK-2). Overall, these data highlighted that renal protection of selective FABP4 inhibitor was substantiated by the reduction of ER stress and inflammation in tubular epithelial cells of rhabdomyolysis-induced injured kidneys and suggested that the inhibition of FABP4 might be a promising therapeutic strategy for AKI treatment.

## Introduction

Rhabdomyolysis is a syndrome characterized by the breakdown of skeletal muscle and leakage of the muscle cell contents such as myoglobin into the bloodstream (Bosch et al., 2009) and acute kidney injury (AKI) is the most common and life-threatening systemic complication of rhabdomyolysis (Raymond et al., 2000). Rhabdomyolysis-induced AKI accounts for ∼15% of all AKI cases (Graham et al., 2004; McMahon et al., 2013). Apart from classical supportive care strategies (e.g., intravenous fluid therapy and renal replacement therapy), the related mortality remains considerably high (Chatzizisis et al., 2008; Zeng et al., 2014).

Although the detailed mechanisms have not been fully comprehended, the endoplasmic reticulum (ER) stress and inflammation played crucial roles in rhabdomyolysis-induced AKI (Panizo et al., 2015; Feng et al., 2016). ER stress has emerged as a major pathophysiological process underlying inflammation and apoptosis (Zhang and Kaufman, 2008). The presence of misfolded proteins and corresponding stress led to the activation of an adaptive program by the ER, known as unfolded protein response (UPR), to restore protein-folding homeostasis (Bird, 2006). Initiation of canonical UPR engaged three distinct signaling pathways, which were regulated by pancreatic ER kinase (PERK), activating transcription factor-6 (ATF6), and inositol-requiring transmembrane kinase/endonuclease-1 (IRE-1) (Ron and Walter, 2007; Hotamisligil and Erbay, 2008). The elicitation of UPR in turn activated c-Jun N-terminal kinases (JNK) and nuclear factor kappa-light-chain-enhancer of activated B cells (NF-κB) pro-inflammatory signaling pathways (Tam et al., 2012). The combined action of the three pathways contributed to the inhibition of protein translation, stimulation of protein degradation and production of chaperone proteins, triggering either recovery of ER function, inflammation or cell death (Zhang et al., 2017).

Fatty acid-binding protein 4 (FABP4), also named as adipocyte P2 (aP2) or adipocyte-FABP (A-FABP), is a novel adipokine involved in energy balance, ER stress and inflammation in diabetes, atherosclerosis, and kidney diseases (Furuhashi and Hotamisligil, 2008; Hotamisligil and Bernlohr, 2015). Under abnormal conditions, FABP4 is over-expressed in adipocytes and macrophages and activates the inflammatory signaling pathways, releasing inflammatory cytokines such as TNF-α, ILs, and monocyte chemotactic factor MCP-1 (Chen et al., 2013). FABP4 as a lipid chaperone also could mediate macrophage ER stress to reduce inflammatory responses and cell apoptosis (Erbay et al., 2009). In human normal kidney tissue, FABP4 is mainly expressed in perianal blood vessels, whereas FABP4 is highly expressed in glomerular CD68-positive macrophages and CD34-positive endothelial cells in lupus nephritis, affecting urine protein and renal function (Tanaka et al., 2014). Previous study also reported that serum FABP4 in AKI patients was positively associated with acute renal dysfunction (Yeung et al., 2009; Ebert et al., 2014; Okazaki et al., 2014).

**FIGURE 1:**
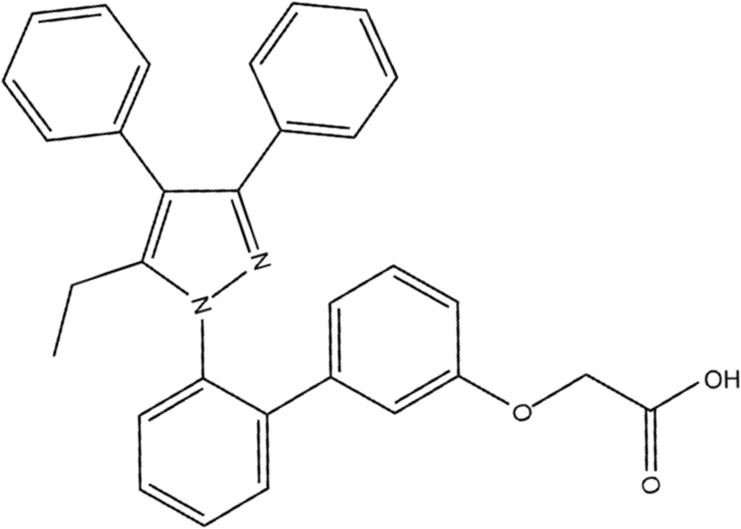
The chemical structure of BMS309403.

In our previous study, overexpression of FABP4 in kidneys was mainly expressed in tubular cells induced by AKI after renal ischemia/reperfusion (I/R) surgery (Shi et al., 2018). Pharmacological inhibition of FABP4 activity by a highly selective inhibitor BMS309403 (**Figure 1**; Furuhashi et al., 2007) resulted in ameliorating renal structural damage, improving renal function and reducing ER stress of tubular cells in mice of I/R-induced AKI. However, the function of FABP4 in rhabdomyolysis-induced AKI remained poorly understood. In the study, chemical inhibition of FABP4 on rhabdomyolysis-induced AKI and the involved mechanisms were investigated.

## Materials and Methods

### Animals and Drug Administration

The animal protocol was approved by the Animal Care and Use Committee of Sichuan University (IACUC number: 2017080A), and all the animal care and experimental procedures were conducted in accordance with The Guide for the Care and Use of Laboratory Animals. Male C57BL/6 mice (8–10 weeks; 25–30 g) were purchased from the Animal Laboratory Center of Sichuan University (Chengdu, China). After 1 week of adaptation to the housing conditions, the mice were randomly divided into three groups (*n* = 10): control, glycerol, and glycerol + FABP4 inhibitor BMS309403. The mice in the latter two groups were injected with 50% glycerol dissolved in 0.9% normal saline (10 ml/kg) at bilateral back limbs to stimulate the rhabdomyolysis-induced AKI model. The same volume of saline was injected in the mice of control group. In the BMS309403 group, BMS309403 (dissolved in 200 μL 30% PEG400) at a dose of 30 mg/kg/d for four consecutive days before glycerol injection. The mice were sacrificed at 24 h after glycerol exposure. Terminal blood samples and kidney tissues were collected for further investigations.

### Renal Function

Serum creatinine levels (SCr) were evaluated by high-performance liquid chromatography, conducted by the Institute of Drug Clinical Trial and the GCP Center of West China Hospital, to assess renal function. Creatine kinase (CK) was measured in the same way.

### Histological Examination

Formalin-fixed, paraffin-embedded kidney sections (4 μm) were stained with hematoxylin and eosin (HE) and periodic acid-Schiff (PAS). Tissue damage was scored on a scale of 0–4, with 0, 1, 2, 3, and 4 corresponding to 0%, <25%, 26–50%, 51–75%, and >76% of injured/damaged renal tubules, respectively. Ten field of ×40 magnification was examined and averaged.

### Western Blotting Analysis

Proteins (**Supplementary Table S1**) were extracted from kidney tissues or HK-2 cells using RIPA buffer containing 4% cocktail proteinase inhibitors and then analyzed by western blotting. Equal amounts of protein were separated by SDS-polyacrylamide gels and then transferred onto a PVDF membrane (Bio-Rad, Hercules, CA, United States). The membranes were incubated with primary antibodies against overnight at 4°C followed by incubation with secondary antibodies (R&D Systems, MI, United States) for 1 h at room temperature. Finally, the proteins were developed with an enhanced chemiluminescence agent (Millipore Corporation, Boston, MA, United States). The signals were measured using an Odyssey Infrared Imaging System (Bio-Rad, ChemiDoc MP, mANUSC, Bio-Rad Laboratories Inc., Hercules, CA, United States) and quantified using the ImageJ program (National Institutes of Health, Bethesda, MD, United States).

### Immunofluorescence Staining

Renal specimens were embedded in optimum cutting temperature (OCT) compound, frozen in acetone dry ice mixture and cut into 3–5 μm sections on a cryostat and stored at -80°C until use. Non-specific binding sites were blocked with phosphate-buffered saline (PBS) containing 5% bovine serum for 1 h at room temperature. For double staining, we incubated the specimens overnight with the first primary antibody at 4°C. After washing with PBS, the corresponding secondary antibody was applied for 1 h. Sections were then incubated overnight with the second primary antibody at 4°C. After washing with PBS, the corresponding secondary antibody was applied for 1 h. The samples were washed with PBS, stained with 4′,6-diamidino-2-phenylindole (DAPI) (Zhongshan Golden Bridge Biotechnology, Beijing, China) and mounted with cover clips. In negative controls, primary antibodies were replaced by PBS. Secondary antibodies (Jackson ImmunoResearch) matched with a corresponding primary antibody were used to display fluorescent signals (1:200 dilution). Confocal images were captured with a Zeiss LSM 710 confocal microscope (Zeiss, Jena, Germany). Images were exported from the ZEN 2012 (blue edition) microscopy software.

### Quantitative Real-Time PCR Analysis

Total RNA from renal tissues was extracted using a total RNA extraction Kit (BioTek, Winooski, VT, United States) according to the protocols. The concentration of mRNA was tested using a Scan Drop 100 (Analytik Jena, Thuringia, Germany) determiner. Quantitative real-time PCR was performed after reverse transcription by using the iQ SYBR Green Supermix (Bio-Rad, Hercules, CA, United States) in a PCR system (CFX Connect; Bio-Rad, Hercules, CA, United States). Relative expression levels were normalized to GAPDH (**Supplementary Table S2**).

### Electron Microscopy

After being fixed in cold 2.5% glutaraldehyde for 2 h at 4°C, kidney tissues were washed with PBS (0.2 mol/L, pH 7.4) for 2 h, fixed with 1% osmic acid for 2 h, and then washed six times with PBS for 10 min per wash. The samples were dehydrated with ethanol and cleaned with epoxypropane. They were embedded in EPON 812 overnight at room temperature. Ultrathin sections (40–60 nm) were cut (EM UC61rt, Leica) and stained with uranyl acetate/lead citrate. These sections were subsequently visualized using a transmission electron microscope (H-7650, Hitachi).

### Cell Culture and Myoglobin Treatment

Human renal proximal tubule cell line (HK-2) was a gift from Prof. Xueqing, Yu (The first Affiliated Hospital, Sun Yat-sen University) and maintained in Dulbecco’s modified Eagle’s medium (DMEM)/F12 (Hyclone, Beijing, China) supplemented with 10% fetal bovine serum (FBS, Hyclone, Australia) at 37°C under humidified atmosphere of 5% CO_2_ and 95% air. We divided the cells in exponential growth state into four groups: the myoglobin group, incubated with 200 μmol/L ferrous myoglobin for 24 h; the Mb-BMS10 group, incubated with BMS309403 at 10 μM 30 min prior to myoglobin treatment; the Mb-BMS30 group, incubated with BMS309403 at 30 μM 30 min before myoglobin treatment; and control group, cells were incubated with complete medium alone in the control group. Ferrous myoglobin was prepared as described previously (Feng et al., 2016; Huang et al., 2017).

### Statistical Analysis

Data were expressed as mean ± standard deviation. Multiple comparisons of means among groups were examined through one-way analysis of variance; a two-sided *P* < 0.05 was considered statistically significant. Between-group comparisons of means were analyzed by least significant difference *t*-tests, and *P*-values were compared to a Bonferroni-corrected significance level of 0.0167 to control the overall significance level for the tests at a family-wise error rate of 0.05. We used SPSS version 19.0 (SPSS Inc., Chicago, IL, United States) for statistical analyses.

## Results

### Glycerol Induced the Upregulation of FABP4 Expression in Kidney of AKI

**FIGURE 2:**
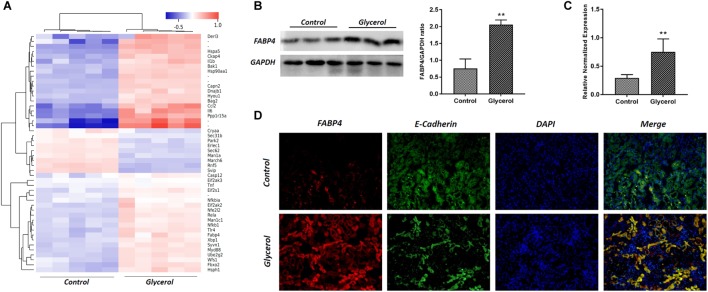
Glycerol-induced the upregulation of FABP4 expression in kidney. **(A)** Hotmap of differentially expressed genes between control and glycerol group. **(B)** The kidney tissue lysates were subjected to Western blotting analysis with indicated antibodies against FABP4 and the expressions of FABP4 was quantified by densitometry and normalized with GAPDH. **(C)** FABP4 mRNA expression in renal tissue was measured by real-time PCR. **(D)** Immunofluorescence staining of FABP4 and E-cadherin in the kidney tissue. E-cadherin was used as a marker of tubular epithelial cells. ^∗∗^*P* < 0.01.

Male C57BL/6 mice were injected by glycerol to create a model of rhabdomyolysis-induced AKI as indicated, and FABP4 protein and mRNA expression was examined. As shown in **Figure 2**, glycerol increased the expression of FABP4 protein. Similarly, the levels of FABP4 mRNA in kidney tissue were markedly increased after glycerol injection. The localization of FABP4 in kidney was confirmed by double immunofluorescence labeling of E-cadherin (green, a biomarker of tubular epithelial cell) and FABP4 (red). Compared with control, increased positive expression of FABP4 was observed in tubular epithelial cells in glycerol-induced AKI group (**Figure 2C**).

### Pharmacological Inhibition of FABP4 Attenuated Glycerol-Induced AKI

**FIGURE 3:**
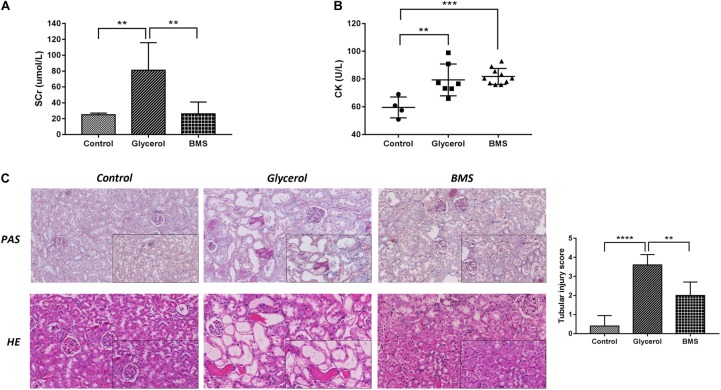
Pharmacological inhibition of FABP4 by BMS309403 alleviated glycerol-induced AKI. **(A)** Serum creatinine (SCr). **(B)** Serum creatine kinase (CK). **(C)** HE/PAS staining of the kidney tissues (×200, ×400). ^∗∗∗∗^*P* < 0.0001, ^∗∗∗^*P* < 0.001, ^∗∗^*P* < 0.01.

To determine whether the inhibition of FABP4 exhibited a renal protective effect, we examined the function of BMS309403, a highly selective small-molecule inhibitor of FABP4, on pathological changes and renal function in glycerol-induced AKI. As demonstrated in **Figure 3**, pretreatment with BMS309403 at a dose of 30 mg/kg/d for four consecutive days decreased the level of serum creatinine without influencing serum creatine kinase change (**Figures 3A,B**). Consistent with improved kidney function by BMS309403 administration, a renal histological examination showed less tubular dilatation, swelling, necrosis, cast formation and preservation of a brush border in the BMS309403-treated AKI group, which was indicated by the quantification of kidney injury score (**Figure 3C**). Taken together, these data suggested that BMS309403 protected against rhabdomyolysis-induced AKI via the inhibition of FABP4 activity.

### BMS309403 Suppressed Renal ER Stress in Glycerol-Induced AKI

**FIGURE 4:**
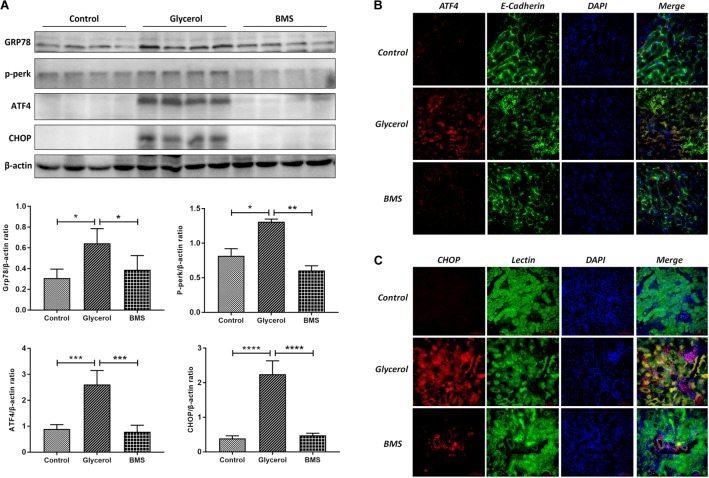
BMS309403 suppressed the expression of ER stress related proteins. **(A)** The expression of GRP78, p-PERK, ATF4, CHOP, as measured by Western blotting analysis, in kidney tissue sections. The densitometry values of proteins were normalized with β-actin. ^∗∗∗∗^*P* < 0.0001, ^∗∗∗^*P* < 0.001, ^∗∗^*P* < 0.01, ^∗^*P* < 0.05. **(B)** Double immunofluorescence staining of ATF4 proteins in tubular epithelial cells. **(C)** Double immunofluorescence staining of CHOP proteins in tubular epithelial cells.

**FIGURE 5:**
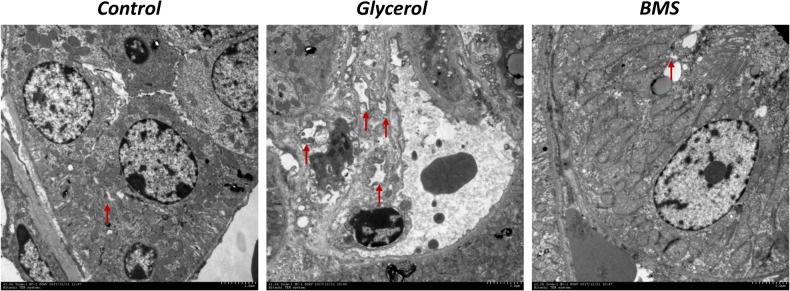
BMS309403 attenuated endoplasmic reticulum expansion in glycerol-induced kidney tissue. Photomicrographs collected by transmission electron microscope (×12 k, red arrow: endoplasmic reticulum).

ER stress contributed to the pathogenesis of rhabdomyolysis-induced AKI. And BMS309403 has been documented to possess the potential to alleviate ER stress in renal I/R injury. We hypothesized that BMS309403 ameliorated rhabdomyolysis-induced AKI through the suppression of ER stress. As illustrated in **Figure 4**, the expression of ER stress-associated proteins, including GRP78, p-PERK, ATF4, and CHOP were significantly upregulated in the kidneys by glycerol injection and suppressed by pretreatment of BMS309403. Furthermore, we performed the immunofluorescence staining of ATF4 and CHOP proteins. The two proteins were minimally expressed in the kidney of control, but remarkably upregulated in that of glycerol group, predominantly located in the renal tubules. Pretreatment with BMS309403 significantly suppressed the expression of ATF4 and CHOP proteins (**Figure 4**). Examination by transmission electron microscopy also has proved the above-mentioned results. In the glycerol group, large amount of swelling ER was observed in the cytoplasm of renal tubular cells, which was not detected in the control group. Oral administration of BMS309403 dramatically reduced the swelling ER in renal tissue of AKI (**Figure 5**). So, these findings indicated that glycerol induced the upregulation of ER-related proteins in tubular epithelial cells, and BMS309403 suppressed FABP4 activity to reduce ER stress in glycerol-induced kidney injury.

### BMS309403 Inhibited Renal Inflammation in Glycerol-Induced AKI

**FIGURE 6:**
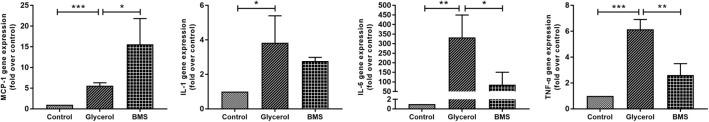
Effect of BMS309403 on glycerol-induced renal inflammation as determined by real-time PCR. Relative expression levels of MCP-1, IL-1β, IL-6, and TNF-α were normalized to GAPDH. ^∗∗∗^*P* < 0.001, ^∗∗^*P* < 0.01, ^∗^*P* < 0.05.

As inflammation played a crucial role in both chronic and acute kidney diseases, we examined the effect of FABP4 inhibition on kidney inflammation after glycerol injection by quantitative real-time PCR analysis. As shown in **Figure 6**, glycerol remarkably upregulated the relative mRNA level of inflammatory cytokines IL-1β, IL-6, TNF-α, and monocyte chemoattractant protein 1 (MCP-1). BMS309403 pretreatment suppressed mRNA expression of IL-6 and TNF-α without influencing the level of IL-1β, while induced MCP-1 mRNA expression.

### BMS309403 Attenuated Myoglobin-Induced ER Stress and Inflammation in HK-2 Cells

**FIGURE 7:**
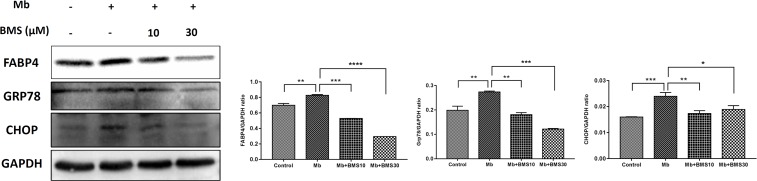
BMS309403 attenuated myoglobin-induced ER stress in HK-2 cells. Expression of FABP4, GRP78, and CHOP proteins were measured by western blot analysis in different groups. The densitometry values of proteins were normalized with GAPDH. Data expressed as means ± SD for groups of three independent experiments. ^∗∗∗∗^*P* < 0.0001, ^∗∗∗^*P* < 0.001, ^∗∗^*P* < 0.01, ^∗^*P* < 0.05.

The pathophysiology of rhabdomyolysis-induced AKI was believed to be triggered by myoglobin as the toxin causing renal dysfunction. We also examined the protective effects of FABP4 inhibitor on the renal proximal tubular cell, the main injury site of rhabdomyolysis-induced AKI, using an *in vitro* myoglobin stimulation. As shown in **Figure 7**, the expression of FABP4 protein in HK-2 cells was significantly increased after 24 h myoglobin stimulation, compared with control. Similarly, the levels of ER stress-associated proteins (GRP78 and CHOP) upregulated in the myoglobin group. When HK-2 cells were co-incubated with BMS309403, myoglobin-induced ER stress was suppressed in a dose-dependent manner.

**FIGURE 8:**
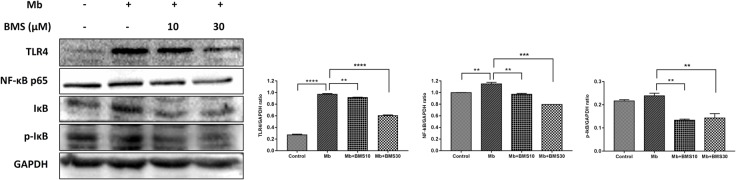
BMS309403 downregulated the myoglobin-induced expression of TLR4/NF-κB in HK-2 cells. Twenty-four hours after myoglobin administration, the expression of TLR4, NF-κB, and p-IκB/IκB were measured by Western blotting analysis. ^∗∗∗∗^*P* < 0.0001, ^∗∗∗^*P* < 0.001, ^∗∗^*P* < 0.01.

Based on our previous study, TLR4/NF-κB pathway regulated renal inflammatory responses in rhabdomyolysis-induced AKI. To determine whether BMS309403 exhibit an effect on TLR4/NF-κB pathway, we compared the expression of TLR4/NF-κB pathway related proteins in different groups. As presented in **Figure 8**, myoglobin induced the upregulation of TLR4, NF-κB p65, and IκB/p-IκB, which was dose-dependently suppressed by BMS309403 administration.

Collectively, these data demonstrated that pharmacological inhibition of FABP4 contributed to ameliorate renal ER-stress and inflammation in tubular cells in rhabdomyolysis-induced AKI.

## Discussion

FABP4 possessed a well-established role in the pathogenesis of diabetes and atherosclerosis (Furuhashi et al., 2007; Erbay et al., 2009), however, the effect of FABP4 in rhabdomyolysis-induced AKI has not been determined. In the present study, we reported pharmacological effect of FABP4 in a murine kidney disease model of glycerol-induced rhabdomyolysis. Selective inhibition of FABP4 by BMS309403 pretreatment at a dose of 30 mg/kg/d for four consecutive days remarkably reduced serum creatinine, proinflammatory cytokine mRNA expression of TNF-α, IL-6, and MCP-1 as well as attenuated tubular damage in glycerol-injured kidneys. Oral administration of BMS309403 attenuated renal ER stress in AKI as evidenced by transmission electron microscope analysis and the related protein expression of GRP78, CHOP, p-PERK, and ATF4. Furthermore, BMS309403 *in vitro* dose-dependently improved myoglobin-induced ER stress and inflammation in renal proximal tubular HK-2 cells.

Rhabdomyolysis is a serious syndrome caused by skeletal muscle injury and subsequent release of breakdown products from damaged muscle cells into systemic circulation (Bosch et al., 2009). The muscle damage often results from strenuous exercise, muscle hypoxia, medications, or drug abuse and commonly leads to life-threatening AKI (Raymond et al., 2000). Myoglobin released from the damaged muscle was believed to trigger renal dysfunction in this form of AKI (Sinniah and Lye, 2000). Although considerable evidences continuously focused on the mechanisms and therapeutic strategy of rhabdomyolysis-induced AKI, the details remained limited. In the study, a well-established animal model of rhabdomyolysis was induced by intramuscular injection of glycerol and further used for the exploration of kidney injury. Although there are several important makers including KIM-1 and NGAL for evaluating renal tubular injuries, sCr and BUN are still the most widely used indices for AKI. Thus, we measured levels of sCr and BUN in animal model of rhabdomyolysis in our study. For the first time, we demonstrated that the mice following glycerol injection developed severe AKI as indicated by acute renal dysfunction and histologic changes, companied by the increased FABP4 expression. Previous study has reported that FABP4 was detected in some tubular cells in patients with stage IV lupus nephritis, not in normal kidneys, suggested that the increase of FABP4 in renal tubular cells might be related with renal dysfunction (Tanaka et al., 2014). In our study, FABP4 was also highly expressed in the cytoplasm of tubular epithelial cells in mice following glycerol injection by double-immunohistochemical staining; consistent with that the increased expression of FABP4 was *in vitro* induced by myoglobin in renal proximal tubular HK-2 cells.

Genetic deletion or chemical inhibition with selective small-molecule inhibitor have been confirmed to have powerful therapeutic for type 2 diabetes and atherosclerosis (Furuhashi and Hotamisligil, 2008). Our previous data indicated that suppression of FABP4 activity by a highly selective inhibitor BMS309403 could ameliorate renal structural damage, improve renal function and reduce ER stress of tubular cells in mice of ischemia/reperfusion-induced AKI (Shi et al., 2018). However, the potential of FABP4 as drug target for the treatment of rhabdomyolysis-induced AKI was still unknown. In the present study, we further investigated and found that pretreatment with BMS309403 effectively reduced serum creatinine and attenuated renal tubular damage in the glycerol-injured kidneys, indicated that pharmacological inhibition of FABP4 improved the kidney dysfunctions in response to rhabdomyolysis. Interestingly, the FABP4 inhibitor BMS309403 protected against kidney injuries without affecting the level of CK, suggested that this inhibitor had no effect on the process of muscle necrosis.

ER stress, an initiator of tubular epithelial injury, could be triggered by different stimuli in AKI, including mutant protein aggregation, hypoxia, energy deprivation, and metabolic dysfunction (Xu et al., 2016; Fan et al., 2017). Declined protein-folding capacity in ER led to an abundance of misfolded proteins, initiating ER stress. Overwhelming ER stress induced inflammatory response by typical signal pathways, PERK-ATF4, IRE1, and ATF6 pathway (Chaudhari et al., 2014). When the ER was overloaded and caused the accumulation of unfolded proteins, GRP78 bonded to the unfolded proteins in the ER, freeing its client proteins PERK, ATF6, and IRE1, which then served as the primary mediators of UPR signaling (Inagi, 2009). During UPR, PERK was released from its chaperone protein GRP78, leading to the activation of ATF4 and CHOP (Gardner and Walter, 2011). The elicitation of UPR in turn activated NF-kB pro-inflammatory signaling pathway to regulate the release of proinflammatory cytokines (Tam et al., 2012). Considerable evidence suggested a link between FABP4 and ER stress in disease models including diabetes, atherosclerosis and kidney disease, etc., (Hotamisligil and Bernlohr, 2015). In our study, we found that along with the high expression of FABP4, the protein levels of ATF4, GRP78, CHOP, in glycerol-injured kidneys of mice and myoglobin-treated HK-2 cells were increased, whereas FABP4 inhibitor BMS309403 decreased the levels of corresponding proteins. No significant changes in expression of IRE1 and ATF6 proteins were found by western blot analysis in our study (data not showed), thus we believe the expression of IRE1 and ATF6 was not mandatory in this study. Taken together, these data indicated that protective effects of BMS309403 against rhabdomyolysis-induced AKI were mediated, at least partially, by the inhibition of renal ER stress.

Proinflammatory cytokines and chemokines are crucial mediators in the pathogenesis of rhabdomyolysis-induced AKI (Jang and Rabb, 2014). Proinflammatory cytokines and chemokines, such as TNF-α, IL-6, and IL-1, an MCP-1 contributed to kidney injury and renal dysfunction (Deshmane et al., 2009; Liu et al., 2018). In the study, the results exhibited that inflammatory cytokines, such as IL-1β, IL-6, and TNF-α were significantly elevated in the kidneys of glycerol-induced mice. Inhibition of FABP4 considerably reduced the production of these inflammatory cytokines, although there was no statistical significance between BMS309403-treated and glycerol group. Our previous results have shown that chemical inhibition of TLR4/NF-κB signal pathway possessed renal protective effects on rhabdomyolysis-induced AKI by regulating proinflammatory cytokines production (Huang et al., 2017), while FABP4 inhibitor BMS309403 dose-dependently improved myoglobin-induced expression of TLR4, NF-κB p65, and p-IκB proteins in renal proximal tubular HK-2 cells, consistent with our previous data. Macrophage infiltration into kidney tissues was responsible for the pathogenesis of rhabdomyolysis-induced AKI in mice (Belliere et al., 2015) and humans (Sinniah and Lye, 2000). MCP-1 is one of the key chemokines that regulate migration and infiltration of monocytes/macrophages to influence immune inflammation (Munshi et al., 2011). Therefore, the elevation of MCP-1 in renal tissue of BMS309403-treated mice might be favorable to macrophage migration to exert anti-inflammatory effects during acute phase. Anyway, further detailed studies are needed to determine whether macrophage FABP4 expression was involved in rhabdomyolysis related kidney injury. These results indicated inhibition of FABP4 affected glycerol-induced kidney injury through regulating inflammatory cytokines production.

ER stress and inflammatory responses are integrated at several levels and modulate each other: inflammation could compromise ER function, and ER stress could promote inflammation (Hotamisligil and Erbay, 2008; Erbay et al., 2009; Bettigole and Glimcher, 2015). The links between inflammatory pathways and ER stress are of great interest in kidney diseases and understanding the intricate links and identification of drug target FABP4 are crucial for developing effective therapeutics against renal disease cluster. However, the intrinsic link between ER stress and inflammatory response should be studied further. In summary, our findings demonstrated that rhabdomyolysis resulted in the increase of FABP4 in tubular epithelial cells and pharmacological inhibition of FABP4 activity by BMS309403 protected against rhabdomyolysis-induced AKI via the regulation of ER stress and inflammation in tubular epithelial cells. Therefore, our data indicated that FABP4 might be a promising target for the treatment of AKI and further investigation of genetic inhibition of FABP4 is in progress.

## Author Contributions

LM and PF conceived and designed the experiments. RH, MS, YuF, FG, JL, LL, YL, JX, SL, and YaF performed the experiments. RH, MS, YuF, LL, and JL analyzed the data. RH and LM wrote the paper. All authors approved the final version of the manuscript.

## Conflict of Interest Statement

The authors declare that the research was conducted in the absence of any commercial or financial relationships that could be construed as a potential conflict of interest.
