# Supplementary material for: Pharmacological Inhibition of Fatty Acid-Binding Protein 4 (FABP4) Protects Against Rhabdomyolysis-Induced Acute Kidney Injury
Source: Front Pharmacol. 2018 Aug 8;9:917. doi: 10.3389/fphar.2018.00917 (PMC6092613; doi:10.3389/fphar.2018.00917)
Supplement: Supplementary file 1 [file Table_1.DOC]

Supplementary Material

# Pharmacological inhibition of fatty acid-binding protein 4 (FABP4) protects against rhabdomyolysis-induced acute kidney injury

Rongshuang Huang^†^, Min Shi^†^, Fan Guo, Yuying Feng, Yanhuan Feng, Jing Liu, Lingzhi Li, Yan Liang, Jin Xiang, Song Lei, Liang Ma^*^, Ping Fu^*^

*** Correspondence:**

Liang Ma
Liang_m@scu.edu.cn

Ping Fu

fupinghx@163.com

# Supplementary Tables

## Supplementary Table 1 Antibody lists

| Name | Company | Catalog Number |
| --- | --- | --- |
| Anti-FABP4 | Abcam, USA | ab92501 |
| Anti-GRP78 | Abcam, USA | ab108613 |
| Anti-DDIT3 (CHOP) | Abcam, USA | ab11419 |
| Anti-TLR4 | Abcam, USA | ab22048 |
| Anti-NF-kB p65 | Abcam, USA | ab13594 |
| Anti-IκBα | Cell Signaling Technology, USA | 4812 |
| Anti-phospho-IκBα | Cell Signaling Technology, USA | 2859 |
| Anti-p-PERK | SANTA CRUZ, USA | sc-32577 |
| Anti-ATF4 | Cell Signaling Technology, USA | 11815 |
| Anti-β-actin | ZENBIO, China | 340042 |
