# Supplementary material for: Pharmacological Inhibition of Fatty Acid-Binding Protein 4 (FABP4) Protects Against Rhabdomyolysis-Induced Acute Kidney Injury
Source: Front Pharmacol. 2018 Aug 8;9:917. doi: 10.3389/fphar.2018.00917 (PMC6092613; doi:10.3389/fphar.2018.00917)
Supplement: Supplementary file 2 [file Table_2.DOC]

Supplementary Material

# Pharmacological inhibition of fatty acid-binding protein 4 (FABP4) protects against rhabdomyolysis-induced acute kidney injury

Rongshuang Huang^†^, Min Shi^†^, Fan Guo, Yuying Feng, Yanhuan Feng, Jing Liu, Lingzhi Li, Yan Liang, Jin Xiang, Song Lei, Liang Ma^*^, Ping Fu^*^

*** Correspondence:**

Liang Ma
Liang_m@scu.edu.cn

Ping Fu

fupinghx@163.com

# Supplementary Tables

## Supplementary Table 2 Primer sequence

| Target | Forward | Reverse |
| --- | --- | --- |
| Mouse FABP4 | 5'-GGGGCCAGGCTTCTATTCC-3' | 5'-GGAGCTGGGTTAGGTATGGG-3' |
| Mouse MCP-1 | 5’-CATCCACGTGTTGGCTCA-3’ | 5’-GATCATCTTGCTGGTGAATGAGT-3’ |
| Mouse IL-1β | 5'-TGGGCCTCAAAGGAAAGAAT-3' | 5'-CAGGCTTGTGCTCTGCTTGT-3' |
| Mouse IL-6 | 5'-ACAACCACGGCCTTCCCTACTT-3' | 5'-CACGATTTCCCAGAGAACATGTG-3' |
| Mouse TNF-a | 5'-ACCCTCACACTCAGATCATCTTC-3' | 5'-TGGTGGTTTGCTACGACGT-3' |
| Mouse GAPDH | 5'-GTATGACTCCACTCACGGCAAA-3' | 5'-GGTCTCGCTCCTGGAAGATG-3' |
